# Social Responsibility: Sustainable Development Goals and COVID-19—Perception Scale of Students from Higher Education Institutions

**DOI:** 10.3390/ijerph19095323

**Published:** 2022-04-27

**Authors:** Pedro Severino-González, Dolores Gallardo-Vázquez, Constanza Ortuya-Poblete, José Romero-Argueta, Efraín Tunjo-Buitrago, Felipe Arenas-Torres, Giusseppe Sarmiento-Peralta

**Affiliations:** 1 Department of Economics and Administration, Faculty of Economics and Social Sciences, Universidad Católica del Maule, Talca 3460000, Chile; 2 Department of Financial Economics and Accounting, Faculty of Economics and Business, Universidad de Extremadura, Av. de Elvas, 06006 Badajoz, Spain; dgallard@unex.es; 3 Undergraduate Business Program, Faculty of Economics and Social Sciences, Universidad Católica del Maule, Talca 3460000, Chile; constanza.ortuyapoblete@gmail.com; 4 Faculty of Sciences and Humanities, Universidad Gerardo Barrios, Usulutan 0614, El Salvador; jesus_romero@ugb.edu.sv; 5 Economics and Critical Thinking Research Group of the Economics, Universidad Colegio Mayor de Cundinamarca, Bogota 110111, Colombia; etunjo@unicolmayor.edu.co; 6 Economics and Humanism of Universidad Santo Tomas, Bogota 110111, Colombia; 7 Accountancy Research Center (ARC), Faculty of Economics and Business, Universidad de Talca, Talca 3460000, Chile; farenas@utalca.cl; 8 Department of Medical Technology, Faculty of Medicine, Universidad Nacional Mayor de San Marcos, Lima 15001, Peru; giusseppe.sarmiento@unmsm.edu.pe

**Keywords:** social responsibility, higher education, university, sustainability, COVID-19, student

## Abstract

Social movements and the consequences of the current health crisis resulting from COVID-19 have deepened social injustices and inequities, which can be addressed through the benchmarks set by the Sustainable Development Goals (SDGs). This research is related to the perspective of Higher Education Institutions (HEIs) as social transformation agents. The purpose of this research is to create a scale to measure students’ perception of the social responsibilities developed by HEIs from the SDGs’ perspective. A matrix solution was found after Exploratory Factor Analysis (EFA) and Confirmatory Factor Analysis (CFA) composed of four dimensions. The constructs that form the four dimensions can be used to design strategies which contribute to the SDGs’ goals, for which it is necessary to have the opinions of the actors that are part of the educational community. Future research should consider carrying out comparative studies according to sociodemographic variables for a better understanding of the social phenomenon.

## 1. Introduction

Social movements are manifestations of groups of people who show their approval or dissatisfaction, as a result of the characteristics of a society [1,2]. These social movements arise from inequalities related to the socioeconomic model [3,4]. Movements around the world are based on causes that may be the product of a regime of government, due to their questioning as guarantors of subsidiary benefits and not of the social rights of citizens [5], as well as humanitarian and environmental causes [6]. In this latter perspective, there are movements that seek to promote the solidarity economy [7] and the dignified treatment of people [8].

The COVID-19 health crisis is a pandemic that has led to the development of initiatives that place the personal and social responsibilities which citizens acknowledge that they have in a position of judgment [9,10], seeking to reduce the number of infections to avoid health systems collapsing. At the same time, it is recognized that health access and coverage must be respected and protected by nations, as it is a fundamental right declared in the Sustainable Development Goals (SDGs) [11]. The solidarity and empathy contribute to avoiding contagions resulting from the high rate of transmissibility, as well as to adequate care seeking to contain the symptoms due to the inexistence of a validated vaccine [12]. That is why ethical and socially responsible decisions can be channeled in the Institutions of Higher Studies [13].

It should be noted that social responsibility is a polysemic and multidimensional concept, which is constructed and configured according to the particularities of the context, territory, perspective, and disciplinary approach [14,15]. There are some consensuses that have remained over time, so it is necessary for us to provide a definition that addresses this research: voluntary commitment that motivates the development of actions that seek to satisfy the needs of the members of HEIs. These actions show values such as solidarity, justice, dignity, and empathy [16].

Regarding the aforementioned points, it is necessary to indicate that the study of social responsibility in times of a pandemic is evidence of the social orientation that HEIs can deliver to influence social, economic, and environmental aspects, which will consider the values that underlie human interactions [17]. Social responsibility is thus a tool that can influence the behavior of university students, encouraging them to respect health recommendations and standards, for which it is essential to implement strategies linked to ethical behavior that contributes to sustainable development, including the health and well-being of society [18].

With this in mind, Higher Education Institutions (HEIs) have the unavoidable responsibility of contributing to professionals’ integral formation, which facilitates future decision-makers with their participation in decision processes. Within this purview, they should address ethical and socially responsible aspects [16], and the recognition of the importance of acting with effective judgment. HEIs can consider the integral formation of citizenship due to their involvement in human development [19]. Nevertheless, research such as that of García Ramos [20] suggests that these practices of social responsibility are incorporated exclusively in the philosophical levels for obtaining certifications or complying with laws and not in the institutional policies that model all the behavior of the same. Rethinking these points, the SDGs allow for quality education for life [21], incorporating values and principles that undergird the skills needed to promote sustainable development [22].

This research allows for reflection on social movements, considering the health crisis from the perspective of the SDGs and from student perceptions of universities’ social responsibilities. This study therefore aims to create a scale to measure students’ perceptions on the social responsibilities developed by HEIs in Chile from the perspective of the SDGs.

This article is structured in the following way after its introduction. The first section theoretically develops the relationship between the SDGs regarding social movements and the COVID-19 health crisis, and the contribution from education to the SDGs. The second section presents the research methodology, addressing the design, participants, instrument, procedures, and strategies of analysis. The third section examines the research results, considering the characteristics of the participants. There is also an Exploratory Factor Analysis and Confirmatory Factor Analysis which were carried out, followed by presenting the new structure of the scale. Finally, the fourth section presents the discussion and conclusion.

### 1.1. Relationship of SDGs with Social Movements

Social movements are the expression of a group of people who demonstrate their support or repudiation in relation to events that are considered transcendental in society [3]. From the perspective of critical thinking, these can be understood as questioning the legitimacy of neoliberalism and the very management of governments [23,24]. This has intensified collective discontent pertaining to social inequalities associated with access to health, education, and public safety, among others [25,26], motivating neglected groups and causing a segregation of groups that are named as minorities [27]. All the above has led to an understanding of the current context through new scenarios. One significant element is the manifestations of groups that fight for environmental causes [28,29].

The protests held in Chile and Colombia are justified by a collective malaise which has led to the development of massive social protests and strong criticism of public and private sector institutions [30], all of which seek equal opportunities for all citizens [31]. Manifestations of this nature, in most cases, are marked by cultural actions, but also by vandalism, which leave people dead, wounded, and maimed, affecting the entire community [32]. It can be noted that the social crisis in Chile has both an internal component [33] and an external component, given that the policies of large economies have cross-sectional effects on the global population [34]. In this aspect, it can be pointed out that the SDGs propose guidelines centered on human rights, peace, and values [35,36], where education should seek to integrate them into each of its objectives and goals [21,37].

### 1.2. COVID-19 Health Crisis and Its Approaches to SDGs

COVID-19, originating from Wuhan, China [38], is a virus characterized by its high rate of transmissibility and by causing cough, body pain, and fever [39]. Research has shown that, due to the absence of a validated vaccine, social distancing measures, confinement, the use of masks, and the use of disinfectant products can contribute to the prevention of this massive contagion that can overwhelm health services [40]. This situation has arisen in several countries around the world, causing chaos and collective questioning.

The pandemic has wreaked havoc on economies and affected various industrial sectors which were already damaged by the prevailing trade war [41], leading to unemployment, increased poverty, and rising crime. It has also accentuated social inequalities and inequities resulting from socio-economic classes. The psychology must contribute to a transformation of habits permitting the development of human behavior centered on social values and socially responsible values [42,43]. The above can be addressed through education in sustainable development since it allows the creation of shared and integral values, which benefits the whole society [21,22].

### 1.3. Higher Education and Its Links to SDGs

Social responsibility has been studied from various contexts, countries, and areas of application, including human rights, principles, values, morals, norms, welfare, and sustainable consumption [13,44]. To a lesser extent, there is research that considers social responsibility and SDGs, the urgency of which is in their links with the development of social awareness, responsibility, and democracy [45,46]. All of the above generates preconceptions favoring the development of a philosophy of life and the construction of an organizational ideology, leading to the definition of policies and decision-making practices based on ethical and shared values [47], which are the consequence of a formation in values resulting from a change in social culture, stimulated by the professional values illuminated in university life [16,48].

The perception of social responsibility in educational institutions shows that, although there are declarations of principles and values, these only serve to project a positive image towards the different stakeholders [16], which are characterized according to the type of interest group and the values that set work environments that can contribute to ethics or corporate social responsibility. The education with a human sense which recognizes that we are interdependent beings, plus the design of strategies based on SDGs, facilitate a genuine and transgenerational praxis [36].

The present study contributes to the inevitable challenge that is inherent to the design of socially responsible strategies in university contexts, whose importance is based on the institutionalization of social responsibility practices [20] and which should include sustainable development plans [49]. It is currently necessary to indicate that social responsibility does not exclusively seek to generate a positive image, but also to contribute to tasks which aid the sustainable development of the educational community, both internally and externally [50].

The United Nations Educational, Scientific and Cultural Organization (UNESCO), in a study analyzing limits through the perspectives of the future of education until 2050 [51], sustains that education must assume an active responsibility to develop human potential. It also mentions well-being and sustainable practices, the need to appropriate interculturality, and indicates the need to create and maintain interconnections. The aforementioned includes the challenges of SDGs, the needs expressed by social movements, and the urgencies that underlie the ravages of the pandemic.

In the same sense, UNESCO published a book explaining the paths that transcend the limit of 2050 [52]. It expressed a set of challenges and questions that invite HEIs to develop an education for all, focused on students, which must consider the determination of topics for the organization of knowledge and the necessary link that HEIs must have with society, the community, and the planet. These connections and recommendations respond to current social demands, where SDGs’ goals, the COVID-19 crisis, and social movements are present.

Nonetheless, Gallardo-Vázquez et al. [53] refer to the theory linked to the general and specific principles and values that, together with other authors, according to Severino-González et al. [16], have tried to identify the basic principles of social responsibility in educational institutions [54,55]. Among them, they highlight: human dignity, human freedom, citizenship and participation, solidarity, environment and sustainable development, and principles and values.

It is significant to reaffirm here that HEIs must contribute to education regarding values, including experience-based strategies, which include concepts such as humanity, sustainability, ethics, and social responsibility. It is necessary in this aspect that all educational actors give support from their spaces to professional development from each of their roles and functions [16,56,57].

Studying social responsibility by SDGs during the pandemic thus facilitates an approach from the behavior of university students, which should motivate HEIs to implement strategies that can take advantage of actions related to dignity, citizenship and participation, and solidarity and freedom [16]. All these things are justified because the understanding of social responsibility is polysemic and therefore installs various challenges which seek to contribute to society from different perspectives, constructs, approaches, and orientations [14]. The preceding points pay tribute to the installation of values underlying university students’ interactions [17,18].

## 2. Materials and Methods

This research is quantitative and cross-sectional in nature [58]. It considers the perception of students and teachers about the social responsibilities developed by HEIs in Chile and Colombia from the perspective of SDGs. The questionnaire proposed by Severino-González et al. [16] is administered here, because it can be used at different educational levels to justify the relevance of this research on the facts associated with social movements and the havoc caused by COVID-19. The next section is divided into the following stages: instrument characteristics, participants, procedures, and analysis strategies.

### 2.1. Instrument Characteristics

The instrument used is divided into three sections. The first section considers a filter question, which permits us to apply the criterion alluding to the research subjects’ main characteristic: are you a student or professor of higher education in Chile or Colombia? The second section considers the HEI social responsibility perception questionnaire, which was designed by Severino-González et al. [16]. This questionnaire was adjusted for its applicability in university contexts and submitted to expert judgment validation, composed of researchers and professors in social responsibility, sustainability, ethics, and higher education who assessed the relevance, clarity, and coherence of each of the dimensions and variables. Table 1 presents the questionnaire with six dimensions: 1. human dignity, 2. human freedom, 3. citizenship and participation, 4. solidarity, 5. environmental and sustainable development, 6. principles and values. Each of the dimensions are composed of variables expressed in affirmation, with responses on a five-level Likert-type scale: strongly disagree (1 point), disagree (2 points), neither agree nor disagree (3 points), agree (4 points), and strongly agree (5 points). These dimensions consider the challenges of the current situation which the planet faces, which shows a set of challenges linked to SDGs, the health crisis, and social demands [52,53].

The third section considers sociodemographic aspects including gender, age, number of family members, territory, and occupation. Finally, only the categories that were chosen according to the identification of each of the research subjects are presented.

### 2.2. Participants

The population was formed by a non-probabilistic sample from Chile and Colombia. The sampling plan is a convenience sample applying the snowball technique. The Chilean sample is composed of 230 students from an HEI belonging to a regional capital city in the central-south zone of Chile with over 200,000 inhabitants, of which 55,627 are university students (2019). Due to the nature of this research, a sample of 292 higher education students from Bogotá (Colombia) was taken, whose population is 7.181 million inhabitants, of which there are 805,214 university students (2020). In each of the applications, the anonymity, security, voluntariness, and confidentiality of the participants were safeguarded. Data collection took place in Chile from October 2019 to January 2020 and in Colombia during September and October 2020. In all cases the applications were online due to the social movements that led to demonstrations and countless marches throughout the country at the end of 2019 [30] as well as the prevailing health crisis in 2020 [38].

### 2.3. Analysis Procedures and Strategies

The data collected were exported, systematized, and organized in a Microsoft Excel spreadsheet, and then processed with the SPSS 18 statistical program. Subsequently, an Exploratory Factor Analysis (EFA) was developed with a sample of students from Chile, considering the principal components extraction method and the Varimax rotated solution [59], which allows for grouping variables and reducing dimensions by constructing factors that constitute the object of study. Next, with the Colombian sample, a Confirmatory Factor Analysis (CFA) was developed which used the statistical and procedural techniques of the formative models, due to the application of statistical tests based on partial least squares. The latter lets us evaluate each latent variable through multiple regressions and factor analysis, which can lead to future elimination of factors depending on the degree of compliance with the quality indicators that are applied at different times in this investigation. Finally, each of the components was reviewed, renamed, and conceptualized.

The sample is made up of 230 students from HEIs in Talca (Chile) and 292 students from HEIs in Bogotá (Colombia). These students, in the case of Chile, form a regional population of 55,627 university students (2019), while in the Colombian case there are 805,214 university students in the Cundinamarca department (2020).

## 3. Results

This section presents the characteristics of the HEI students, as well as the EFA and CFA. The latter was developed through partial least squares (PLS), because through variance analysis, it lets us increase the explanatory capacity of the empirical verification of the theory through simultaneous regressions. The new structure of the scale is then presented.

### 3.1. Characteristics of the Participants

Table 2 shows the sociodemographic data collected through applying the survey in Chile and Colombia, with 522 participants. In Chile, there were slightly more males, at 53%. Regarding age group, the highest category was those between 20 and 24 years old, at 75.7%. Regarding family group size, 49.5% said they had between three and four people. For territory, 71.7% declared that they came from an urban area. In Colombia, in terms of gender, more males participated, at 63.7%. Regarding the age group, the highest category once again was those between 20 and 24 years old, with 71.6%. Regarding family group size, the majority said they had between three and four people, totaling 63.7%. Finally, for territory, 95.5% declared that they came from a rural area.

### 3.2. Exploratory Factor Analysis (EFA)

Given the space available in the literature on HEIs’ social responsibility from the perspective of the SDGs and its approach from the social movements and the COVID-19 health crisis, it was decided to design a questionnaire based on the proposal of Severino-González et al. [16], giving way to its validation through the EFA. This statistical technique permits variable grouping and reduction in dimensions that are correlated through the construction of factors.

It is necessary to indicate that for this type of research it is important to have a large sample size, maximizing the reduction in the sampling error. However, for the adequate development of the EFA, the number of participants must be high. Nonetheless, for this exploration, the number of participants is 230 students, which allows a moderate classification [59].

In order to verify proper EFA application, the Kaiser–Mayer–Olkin (KMO) sample adequacy measure was first examined. This measure determines the quality of the data, so that values higher than 0.6 would indicate that the data have an appropriate quality and the EFA can be carried out. Bartlett’s sphericity test, which manifests the absence of correlations between variables, can also be carried out, in which case the significance value should be less than 0.100, inferring that the data are adequate for the analysis [60]. The KMO value is 0.900, while the values associated to Bartlett’s sphericity test are Chi2 = 2721.024; gl = 231; *p* < 0.000. All the above lets us infer that the matrix of variables is factorable and the EFA is appropriate.

### 3.3. Main Component Extraction Method

After verifying compliance with the statistical criteria for EFA application, it is used as a method to extract principal components, considering a rotated solution through the Varimax technique, because it “seeks to redistribute the variance along all the components in the load matrix. With this, the model is simplified, and clearer results are obtained...” [61] (p. 286).

To select the variables that integrate the respective dimensions, the saturation value associated with the vector greater than 1.0 is used as a criterion, leaving out all the variables that have lower values. However, the total variance explained is 61.686%.

### 3.4. Identification of Rotated Components

Once the factors have been extracted, the procedure associated with the Varimax rotation technique is applied because analyzing it facilitates the interpretation of the results. In Table 3, the matrix of rotated components shows that the value of each factor is greater than 0.578, which is higher than the recommended minimum of 0.55 [62]. The composition of each factor that configures each dimension is as follows: 1. freedom and citizenship nine items), 2. empathy and solidarity (five items), 3. respect and dignity (five items), and 4. environment (three items). Finally, the variables P1, P2, P3, P4, and M4, which do not meet statistical, theoretical, and empirical criteria, are left out [63].

### 3.5. Confirmatory Factor Analysis

The Confirmatory Factor Analysis was developed with a Colombian sample. A formative measurement model was used, because each indicator represents a dimension of the meaning of the latent variable and eliminating one of them makes it lose its meaning [64]. All this allows testing and measuring the proposed scale after the development of the EFA [65]. For this, it is necessary to use techniques based on structural equation models for which the Smart PLS v.3.2.8 software was used. The techniques used in this study are discriminant validity, multicollinearity assessment through Variable Inflation Factors (VIF), goodness-of-fit index, and bootstrap-based exact fit test.

Firstly, multicollinearity (VIF) was evaluated, with all cases being less than 2.117. This is below the maximum acceptable except for item D2, L4 and S1, the latter being eliminated because it contributes to the factorial solution [66]. In correspondence to the normalized square root residual index (SRMR), the value found is 0.069, which is considered satisfactory since it is below the limit indicated by Huand Bentler [67]. Regarding the proposal of Henseler et al. [68], for the development of the analysis of the fit of the saturated model and the measurement model, it is developed through bootstrapping, where d_ULS = 0.718 and d_G = 0.262, which are below the 95th percentile and allows us to infer that there are no significant discrepancies.

Subsequently, it is necessary to examine the reliability and convergent validity. Regarding the development of the reliability analysis, the internal consistency has been determined, while each of the composite reliabilities was estimated since, due to this inquiry, it is more appropriate to develop the analysis with the latter indicator, since it is considered insufficient [69]. In this sense, composite reliability was used because it estimates the extent to which sets of indicators of latent constructs share the measure of a construct, and the analysis of composite reliability allows pointing out that all indicators are reliable [66], the lowest being 0.744 (see Table 4).

Next, the co-regulatory validity is examined, which evaluates the degree of interrelation between each of the constructs that were observed through the average variance extracted (AVE). According to Fornell and Larcker [70], the values of each of the factors must be greater than 0.5. The lowest is 0.574, corresponding to the liberty and citizenship factor, which allows this quality criterion to be exceeded (see Table 4). These results are consistent with the challenges pointed out by Sabzalieva et al. [52], since it invites the development of actions addressing the challenges of SDGs, the ravages of the pandemic, and the social demands of various communities worldwide. This is linked to the constructs anticipated in the research of Severino et al. [16] and stressed through other research that has similar characteristics from the approach, subject of study, or context [13,44,53].

Discriminant validity was then examined through PLS analysis in order to determine the differences between each of the constructs found [71]. This is because the use of least squares (PLS) makes it possible, through quality criteria, to verify the suitability of the variables and dimensions, stressing the theory with empirical data.

In this context, it should be noted that after applying the Fornell and Larcker [70] criterion, items S2, L2, and C4 were eliminated. This allowed us to demonstrate that the variances between the items of the same factor are greater than the variances between the factors of other factors [72]. Table 5 shows that the quadratic root of the AVE (value on the diagonal) is higher regarding the other factors that integrate the model (0.767 > 0.546, 0.687 and 0.753; 0.878 > 0.546, 0.544 and 0.602; 0.772 > 0.687, 0.544 and 0.741; 0.788 > 0.753, 0.602 and 0.741). All of the above allows us to affirm that there is discriminant validity [70].

The questionnaire initially proposed after the development of the EFA and the Confirmatory Factor Analysis (CFA) is composed of four factors. Freedom and citizenship is composed of six indicators; factor two, environment care, is composed of three indicators; factor three, respect and dignity, is composed of four indicators; and factor four, empathy and solidarity, is composed of three indicators (see Table 6). The results obtained are similar to what was found in Severino et al. [16] and different when compared to Gallardo-Vázquez [44], which may be due to the context [13,19] or due to incorporating new theories or constructs [53].

## 4. Discussion

Social responsibility in higher education is highly important due to its social impact, which is linked to the role of HEIs as protagonists or forgers of transcendental changes in society, determining in turn the development of strategies that contribute to social conscience [73,74]. All of the above is directly related to SDG 4, since quality education is responsible for developing skills that lead to the understanding of injustices, disparities, and violence as practices that threaten human integrity and dignity (SDG 16), which has become more relevant due to the social movements that have taken place in recent years [26].

The variables corresponding to the dimension of freedom and citizenship must be considered for the design of strategies that seek to promote free expression and respect among community members. In a similar vein, Domínguez and López [75] point out that HEIs are socially responsible when they are able to show society—through an open dialogue—new guidelines that avoid undesired situations [76]. This final point is related to the variables belonging to the empathy and solidarity dimension, a dimension that is associated with SDGs 1 and 2—since poverty and hunger have a relation of interdependence—which is a product of the actions carried out by HEIs that seek to help people with few resources and to integrate all the members of the community.

The variables related to the respect and dignity dimension contribute to the implementation of improvement strategies, which could more robustly promote an environment of respect and healthy relationships among members of the institution. All of the above is part of the demands expressed by social groups that have demonstrated their collective discontent in Chile, which is associated with causes linked to health, education, equality, and inclusive progress (SDGs 3, 4, 5, and 8). A similar situation occurs regarding the evaluation of the environment care dimension; in this aspect, initiatives could be implemented which stimulate the care of nature from its curricular formation (SDG 4), adopting as case studies the environmental problems of the community (SDGs 6, 11, 13, 14 and 15), since these demands are reasons for social manifestations [29].

Gallardo-Vázquez et al. [53] consider social responsibility from university management through actions that pay tribute to students’ needs, where activities are proposed including values training, integrating students with disabilities, and the promotion of self-employment (SDGs 4, 8 and 11). Instead, this research addresses the social responsibility of HEIs from the formation that underlies the strategies that constitute healthy environments which promote learning in ethical principles and values (SDGs 3, 11, 12 and 16). Instead, Gallardo-Vázquez [44] considers the students’ perception of actions related to competition, training, and participation that seek to pay tribute to the needs of various interest groups.

Severino et al. [13] raises the issue of university students’ social responsibility, which shows the degree of commitment to others and the environment. This includes the formation of social responsibility, approach to exercising social commitment, and personal discovery of values (SDSs 12, 15 and 13). Instead, this research considers the strategies of HEIs for training in values that transcend the exclusive training in the classroom, which contributes to the training of competent and socially responsible professionals [77].

## 5. Conclusions

The study of social movements and the inquiries about COVID-19 have taken on greater force in recent times, as they seek to explain the phenomena motivating the development of said action. Research on COVID-19 seeks to find a strong medicine to alleviate the impact on human health and, subsequently, to identify the effects of COVID-19 on society. Both situations can be understood in a convergent way through explorations of social responsibility from the perspective of human behavior and its perceptions.

In this context, this article, through its findings that account for the exploration of students’ perception of social responsibilities developed by HEIs in Chile and Colombia, allows the identification of constitutive strategies implemented by the HEIs that account for actions related to social responsibility. In this sense, it is necessary that these actions can apply the SDGs, especially those related to education, health, environment, and equality. All the above allows us to contribute to the generation of social transformations that seek a balanced and fair development for all.

The results of this research let us suggest that HEIs can implement strategies which contribute to creating environments that instill values such as empathy, solidarity, justice, and transparency. In relation to this context, plans and programs can be designed according to the variables identified in each of the dimensions proposed by this research, including: freedom and citizenship, care for the environment, respect and dignity, and empathy and solidarity. These points make it possible to meet the needs of students, including practices of social responsibility, SDGs’ goals, and the urgencies manifested through social movements.

SDGs install various challenges evidenced in this investigation, which are related to the practices that HEIs can implement. This pays tribute to the demands of society while also allowing students to install skills and values leading to the recognition of professional practice as an action that does not stem from the problems present in the community. These show values that contribute mainly, according to the findings of this research, to the points related to SDGs 3, 4, 5, 6, 11, 13, 14, and 15.

The findings of this research contribute to the theoretical discussion on social responsibility and its links with the SDGs, in correspondence to the values that can be formed in the teaching and learning processes. It is this scenario, orientations, directives, and strategic programs can be developed which allow the implementation of practical actions that could respond to the demands expressed by social movements which have been installed through various demonstrations.

It is necessary to indicate that the present study has some limitations that can be solved in future investigations. The first is the sampling process, as the sample is not probabilistic, which does not allow its easy comparison with other results and, above all, its generalization. Another limitation is the data analysis strategy, since it is necessary to use other statistical tests that allow for more findings. Finally, it is necessary to apply the instrument to diverse HEI interest groups for its comparison and the determination of causes and effects of the perceptions.

However, it can be pointed out that the main contribution of this article is providing information that could eventually be used for the design of strategic orientations by HEIs. This could lead to the development of focused actions that address the most downward aspects of social responsibility. In addition, the entire text seeks to generate connections between social responsibility, SDGs, COVID-19, and social movements.

It is also relevant that future research may consider different stakeholders, which could allow a comparative study between internal and external stakeholders. In addition, it is suggested to develop medium-term research, to explore post-pandemic and post-social-movement effects. Finally, it would be interesting to develop comparisons with the realities of different countries in Latin America and the Caribbean, since the cultural and territorial context can be a determining factor in terms of assessing HEIs’ social responsibility strategies.

## Figures and Tables

**Table 1:** Questionnaire on HEI (Higher Education Institution) social responsibility perception.

Dimension	Items/Affirmation
Human Dignity	D1. There is an atmosphere of respect among the members of the institution.
	D2. Good interpersonal relationships are fostered.
	D3. Personal and emotional support is given to members who present some difficulty.
	D5. Resources are invested to develop activities in minimum conditions of hygiene and security.
Human Freedom	L1. Spaces are provided for the free expression of ideas and/or beliefs.
	L2. The decisions agreed upon by the various competent authorities are respected.
	L3. The ideas and initiatives of the people who are part of the institution are welcomed.
	L4. Spaces for conversation about problems affecting society and the environment are promoted.
Citizenship and Participation	C1. Mechanisms are available to raise opinions and concerns of institutional members.
	C2. Their opinion is considered in the definition of tasks and responsibilities.
	C3. The HEI contributes to the formation of a solid opinion on issues that affect the community.
	C4. Respect for institutional members’ rights and duties is promoted.
	C5. Institutional members are incorporated into various social activities.
Solidarity	S1. Support for communities with limited resources is promoted, as well as for excluded, vulnerable and/or minority groups.
	S2. Respectful treatment of all persons without exception (ethnicity, disability, gender, sexual orientation, etc.) is promoted.
	S3. Activities integrating all institutional members are carried out.
	S4. The necessary importance is given to consider the reality of another member of the institution.
Environment and sustainable Development	E1. Education is given about preventing diseases which affect its members’ integrity.
	E2. The care of the environment is included in the curricular formation.
	E3. The correct use of water, energy and gas is promoted.
	E4. There are containers to separate garbage according to material type (glass, paper, plastic, organic waste, etc.).
	E5. Awareness of environmental problems affecting society in general is created.
Principles and Values	P1. The HEI acts under principles and values generally accepted by the community.
	P2. The authorities demonstrate coherence between their actions and declared principles.
	P3. Sanctions are available for dishonesty, discrimination, and unethical behavior.
	P4. Fulfilling the stated commitments is promoted among institutional members.

Source: Severino-González et al. [16].

**Table 2:** Participant characteristics.

Country	Variables	Categories	Values (%)
Chile	Gender	Female	47
		Male	53
	Age	15–19	13
		20–24	75.7
		25–29	8.7
		30–35	2.6
		Above 35	0
	Number of family members	1–2	20.5
		3–4	49.5
		Above 5	30
	Territory	Urban	71.7
		Rural	28.3
Colombia	Gender	Female	36.6
		Male	63.7
	Age	15–19	12
		20–24	71.6
		25–29	12.3
		30–35	3.4
		Above 35	0.7
	Number of family members	1–2	13.1
		3–4	63.7
		Above 5	23.2
	Territory	Urban	4.5
		Rural	95.5

**Table 3:** Matrix of rotated social responsibility components in HEIs from the SDG (Sustainable Development Goals) perspective.

Variable	Component			
	Freedom and Citizenship	Empathy and Solidarity	Respect and Dignity	Environment
L4	0.727			
C2	0.724			
C3	0.668			
L3	0.661			
L2	0.659			
C4	0.632			
C1	0.631			
L1	0.600			
C5	0.578			
S2		0.734		
S4		0.724		
S1		0.667		
S3		0.605		
E1		0.591		
D2			0.750	
D1			0.740	
D4			0.707	
D3			0.668	
D5			0.588	
E3				0.843
E5				0.794
E2				0.761
Alfa	0.900	0.820	0.820	0.830

**Table 4:** Confirmatory Factor Analysis, reliability analysis, and convergent validity.

Items	Factor	Charges (λ)	Cronbach’s Alpha	rho A	Compound Reliability	AVE *
C1	Liberty and citizenship	0.785	0.851	0.853	0.89	0.574
C2		0.757				
C3		0.736				
C5		0.78				
L1		0.76				
L3		0.724				
E2	Environmental care	0.883	0.845	0.848	0.907	0.764
E3		0.858				
E4		0.881				
D1	Respect and dignity	0.785	0.771	0.775	0.853	0.592
D3		0.793				
D4		0.768				
D5		0.731				
E1	Empathy and solidarity	0.781	0.744	0.754	0.855	0.664
S3		0.772				
S4		0.887				

* Average variance extracted.

**Table 5:** Discriminant validity.

	Empathy and Solidarity	Environment Care	Freedom and Citizenship	Respect and Dignity
Empathy and Solidarity	0.767			
Environment Care	0.546	0.878		
Liberty and Citizenship	0.687	0.544	0.772	
Respect and Dignity	0.753	0.602	0.741	0.788

**Table 6:** Final indicators that make up the scale of social responsibility of HEIs from the perspective of SDGs according to the CFA.

Factors	Indicators	Assertions
Freedom and Citizenship	C1	Mechanisms are in place to raise institution members’ opinions and concerns.
	C2	Your opinion is considered in the definition of tasks and responsibilities.
	C3	It contributes to the formation of a solid opinion on issues that affect the community.
	C5	Respect for institutional members’ rights and duties is encouraged.
	L1	Members of the institution are involved in various social activities.
	L3	The ideas and initiatives of institutional members are welcomed.
Environment Care	E2	Care for the environment is included in the curriculum.
	E3	The correct use of water, energy and gas is encouraged.
	E4	Deposits are available for separating garbage according to type of material (glass, paper, plastic, organic waste, etc.).
Respect and Dignity	D1	There is an atmosphere of respect among the members of the institution.
	D3	Personal and emotional support is provided to members who are experiencing difficulties.
	D4	Respect for people who are not part of the institution is encouraged.
	D5	Resources are invested to carry out activities under minimum hygiene and safety conditions.
Empathy and Solidarity	E1	They are educated in the prevention of diseases which affect their members’ integrity.
	S3	Respectful treatment of all people without exception (ethnicity, disability, gender, sexual orientation, among others) is promoted.
	S4	Activities that integrate all members of the institution are carried out.

## Data Availability

The data presented in this study are available from the corresponding author on reasonable request.
